# Carboxyhemoglobin levels in medical intensive care patients: a retrospective, observational study

**DOI:** 10.1186/cc11138

**Published:** 2012-01-11

**Authors:** Andreas S Fazekas, Marlene Wewalka, Christian Zauner, Georg-Christian Funk

**Affiliations:** 1 Department of Respiratory and Critical Care Medicine, Otto Wagner Hospital, Sanatoriumstrasse 2, A-1140 Vienna, Austria; 2 Ludwig Boltzmann Institute for Chronic Obstructive Pulmonary Disease and Pneumologic Epidemiology, Otto Wagner Hospital, Sanatoriumstrasse 2, A-1140 Vienna, Austria; 3 Department of Internal Medicine III, Gastroenterology and Hepatology, General Hospital Vienna, Währinger Gürtel 18-20, 1090 Vienna, Austria

## Abstract

**Introduction:**

Critical illness leads to increased endogenous production of carbon monoxide (CO) due to the induction of the stress-response enzyme, heme oxygenase-1 (HO-1). There is evidence for the cytoprotective and anti-inflammatory effects of CO based on animal studies. In critically ill patients after cardiothoracic surgery, low minimum and high maximum carboxyhemoglobin (COHb) levels were shown to be associated with increased mortality, which suggests that there is an 'optimal range' for HO-1 activity. Our study aimed to test whether this relationship between COHb and outcome exists in non-surgical ICU patients.

**Methods:**

We conducted a retrospective, observational study in a medical ICU at a university hospital in Vienna, Austria involving 868 critically ill patients. No interventions were undertaken. Arterial COHb was measured on admission and during the course of treatment in the ICU. The association between arterial COHb levels and ICU mortality was evaluated using bivariate tests and a logistic regression model.

**Results:**

Minimum COHb levels were slightly lower in non-survivors compared to survivors (0.9%, 0.7% to 1.2% versus 1.2%, 0.9% to 1.5%; *P *= 0.0001), and the average COHb levels were marginally lower in non-survivors compared to survivors (1.5%, 1.2% to 1.8% versus 1.6%, 1.4% to 1.9%, *P *= 0.003). The multivariate logistic regression analysis revealed that the association between a low minimum COHb level and increased mortality was independent of the severity of illness and the type of organ failure.

**Conclusions:**

Critically ill patients surviving the admission to a medical ICU had slightly higher minimum and marginally higher average COHb levels when compared to non-survivors. Even though the observed differences are statistically significant, the minute margins would not qualify COHb as a predictive marker for ICU mortality.

## Introduction

CO is synthesized naturally in the body and serves a range of physiological functions including vasodilation, angiogenesis, vascular remodeling, protection against tissue damage and modulation of the inflammatory response [1,2]. Approximately 85% of the CO is produced by heme oxygenase (HO), which catalyses heme to CO, iron and biliverdin. Biliverdin is further broken down into bilirubin [3]. The major site of heme catabolism, and thus CO production, is the liver [4]. The normal blood COHb saturation in non-smokers is approximately 1% [5], the mean saturation in smokers of approximately 20 cigarettes per day lies around 5.5% [6]. The majority of CO is removed from the body via expiration [7]. Of the two isoforms of heme oxygenase (HO-1, HO-2), HO-1 is the only inducible isoform [8]; it is induced by oxidative stress, hypoxia, heavy metals, sodium arsenite, heme and heme derivatives, as well as by cytokines [9-11].

Increased expression of HO-1 and elevated COHb levels have been demonstrated in patients with critical disease, chronic obstructive pulmonary disease (COPD), systemic inflammatory response syndrome and acute respiratory distress syndrome [12-14]. HO-1 induction may be beneficial because its products possess anti-inflammatory and antioxidant properties [15,16]. However, excessive HO-1 activity is deleterious, possibly due to the liberation of molecular iron [17]. Melley *et al. *observed that patients who were admitted to an ICU following cardiothoracic surgery were more likely to die in the ICU if they had lower minimum or higher maximum COHb levels [18], thus supporting the hypothesis that there is an optimal range for HO-1 induction [17]. Of interest, inhaled CO is also currently being tested as a therapeutic agent based on evidence of cytoprotective and anti-inflammatory effects from animal studies. The observed peak levels of COHb in these preclinical studies typically range from 5% to 30% [19]. However, the therapeutic potential of CO in humans is limited by its toxicity. For example, even low levels of 2% to 6% COHb decrease exercise time to angina or produce an increase in arrhythmias in non-smoking patients with known coronary artery disease [20,21]. On the other hand, healthy volunteers have been shown to tolerate levels of 12% to 14% without serious side effects [19].

We proposed the following hypothesis for our study: because abnormal COHb levels correlate with an increased ICU mortality in critically ill medical patients, COHb would serve as a predictive marker for ICU mortality. In addition, detailed knowledge about the characteristics of COHb levels in critically ill patients would be of use for experimental studies involving CO as a therapeutic agent.

## Materials and methods

### Study population

The study was observational and retrospective in nature. All non-surgical patients who were consecutively admitted to one of the ICUs of the General Hospital of Vienna between December 3, 2001 and September 26, 2005 were considered for inclusion. Because no additional interventions were undertaken and analysis was performed on anonymous data, the need for informed consent was waived. The local ethics committee approved the study. The exclusion criteria included previous admissions to the ICU, planned withdrawal of therapy within 24 hours, and a surgical cause for admission.

### Data collection

Results from point of care analyzers for every patient who was admitted to the ICU were automatically downloaded into a computerized clinical information system. Collected data were manually checked for accuracy and were linked to the patients' medical record numbers. The admitting physician manually recorded the reason for admission in the patient files. Patients were assigned to a distinct admission category based on the recorded reason for admission [22].

### Laboratory assays

COHb levels were determined immediately upon ICU admission and every four to six hours thereafter using a heparinized blood sample that was collected from an indwelling arterial catheter. The point of care analyzer runs a zero calibration of the optical system against a colorless calibration fluid at least every four hours to guarantee accuracy.

### Severity of illness and ICU outcome

Severity of illness was assessed by the Simplified Acute Physiology Score II (SAPS II) [23]. Scoring was performed on the worst values that were recorded in the 24 hours following admission to the ICU using the data retrieved from the computerized clinical information system. The outcome variables measured included ICU mortality and ICU length of stay. Furthermore, the presence of organ failure in the respiratory, circulatory, renal, hepatic, and cerebral system was recorded based on Sepsis-related Organ Failure Assessment (SOFA) criteria [24].

### Data analysis

Data are presented as median and interquartile ranges or as mean and standard deviation. Minimum, maximum, average and area under the curve values as well as the variance for measured COHb levels were calculated according to the Melley study [18]. Statistical tests and mathematical modeling were performed using PASW v18. Correlation in normally distributed variables was calculated by Pearson's coefficient. Multivariate logistic regression was performed using the vital status upon ICU discharge as the response variable and the COHb level as the primary exposure variable. Predefined confounding variables were included in the initial regression model, including the SAPS II score, the admission category, the presence and type of organ failure, and the age and gender of the patients. Models were subsequently refined by backward exclusion.

To compare serial COHb measurements according to ICU survival, we used a generalized estimating equation assuming a first order exponential correlation matrix for repeated observations within one patient.

For all analyses statistical significance was defined by a two-sided *P *< 0.05.

## Results

### Admission characteristics

**Figure 1:**
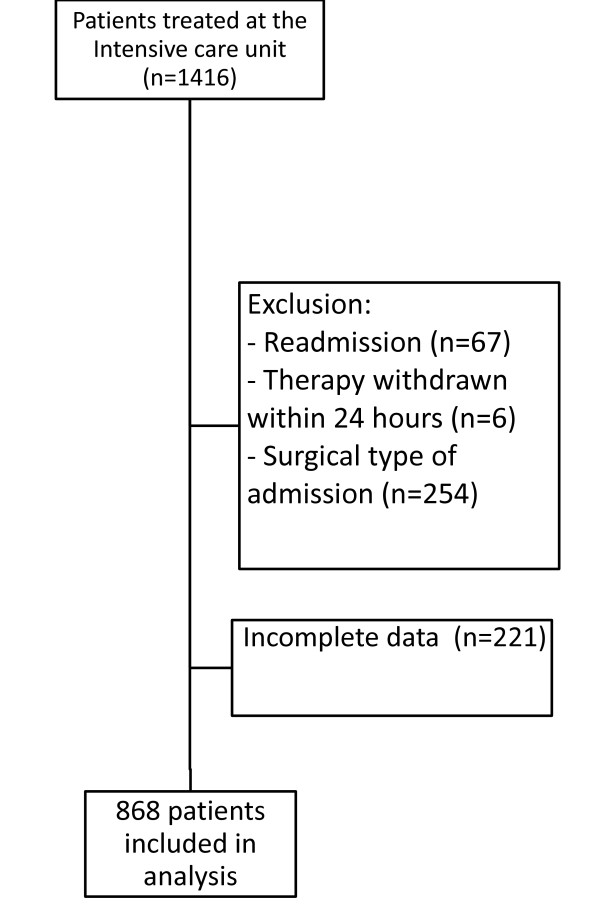
**Study Flow Chart**.

The study flow chart is presented in Figure 1. A total of 1,416 patients (1,483 admissions) were admitted to the ICU during the 46-month study period. The analysis was restricted to the first admission for the 67 patients with multiple ICU admissions; 481 patients were excluded from the analysis (Figure 1).

The causes of admission and the types of organ failure in the remaining 868 patients are shown in Table 1. There were 525 (60%) male patients, and the median age of the patients was 59 years old (47 to 70). The median SAPS II score was 49 (34 to 62). The median length of ICU stay was 6 days (3 to 11), and 220 patients (25%) died in the ICU. SAPS II scores were significantly higher in the group of non-survivors. There was no difference in age between the two patient groups. Circulatory, renal, hepatic, and cerebral failure was significantly and independently associated with ICU mortality, whereas respiratory failure did not show a correlation (Table 2).

**Table 1:** Admission category and organ failure on admission in the entire cohort.

Admission Category	Number	Percent (%)
Metabolic disease	4	0
Respiratory disease	234	27
Cardiovascular disease	204	24
Shock	6	1
Renal disease	23	3
Neurologic disease	74	9
Sepsis	80	9
Trauma (not operated)	3	0
Gastrointestinal disease	109	13
Hematologic disease	20	2
Medical, not otherwise specified	108	12
Pregnancy	3	0
**Organ Failure on admission**		
Respiratory	638	74
Circulatory	454	52
Renal	270	31
Hepatic	204	24
Cerebral	373	43

Organ failure was defined according to SOFA criteria [24]

**Table 2:** Admission category and organ failure according to the survival status.

		ICU survivors (n = 648)		ICU non-survivors (n = 220)		
		Number	%	Number	%	
**Admission Category**	Metabolic disease	4	1	0	0	
	Respiratory disease	196	30	38	17	
	Cardiovascular disease	155	24	49	22	
	Shock	2	0	4	2	
	Renal disease	21	3	2	1	
	Neurologic disease	61	9	13	6	
	Sepsis	44	7	36	16	
	Trauma (not operated)	2	0	1	0	
	Gastrointestinal disease	75	12	34	15	
	Hematologic disease	14	2	6	3	
	Medical, not otherwise specified	71	11	37	17	
	Pregnancy	3	0	0	0	
						*P*-value
**Organ Failure**	Respiratory	472	73	166	75	0.45
	Circulatory	290	45	164	75	0.0001
	Renal	158	24	112	51	0.0001
	Hepatic	124	19	80	36	0.0001
	Cerebral	265	41	108	49	0.034

### COHb levels in the entire cohort

The mean COHb level in all patients was 1.6% (1.3% to 1.9%), which was elevated compared to healthy non-smokers (approximately 0.8%) but considerably less than the COHb levels in current smokers (approximately 6% to 8%) [5]. The minimum and maximum COHb levels were 1.1% (0.8% to 1.4%) and 2.1% (1.7% to 2.6%), respectively, in each patient. The complete characteristics of the COHb levels including the variance and area under the curve results are shown in Table 3.

**Table 3:** COHb in the entire cohort, values expressed as % of total hemoglobin

	Mean	Standard-deviation	Median	Percentile 25	Percentile 75
**COHb minimum**	1.1	0.5	1.1	0.8	1.4
**COHb maximum**	2.2	0.8	2.1	1.7	2.6
**COHb mean**	1.6	0.5	1.6	1.3	1.9
**COHb variance**	0.1	0.3	0.1	0	0.1
**COHb area under curve**	1.6	0.5	1.6	1.3	1.9

AUC, area under the curve.

### COHb levels according to admission category and severity of illness

**Figure 2:**
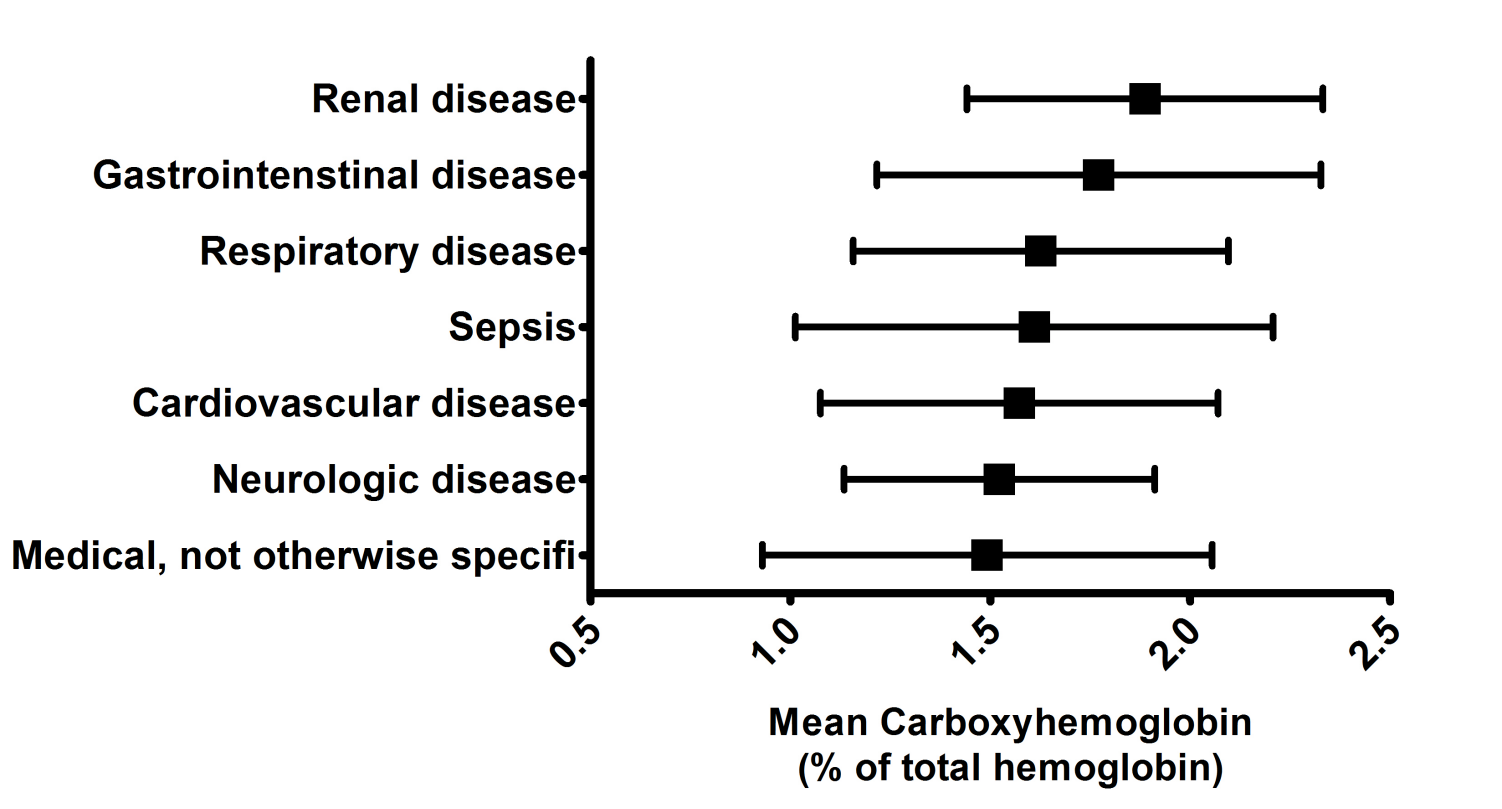
**Mean carboxyhemoglobin during the stay on the ICU according to the admission category**. *P *< 0.0001 in univariate ANOVA.

Mean COHb differed between admission categories (Figure 2). Highest values were observed in patients with renal and gastrointestinal disease. Lowest levels were seen in the neurological and medical (not otherwise specified) categories.

**Figure 3:**
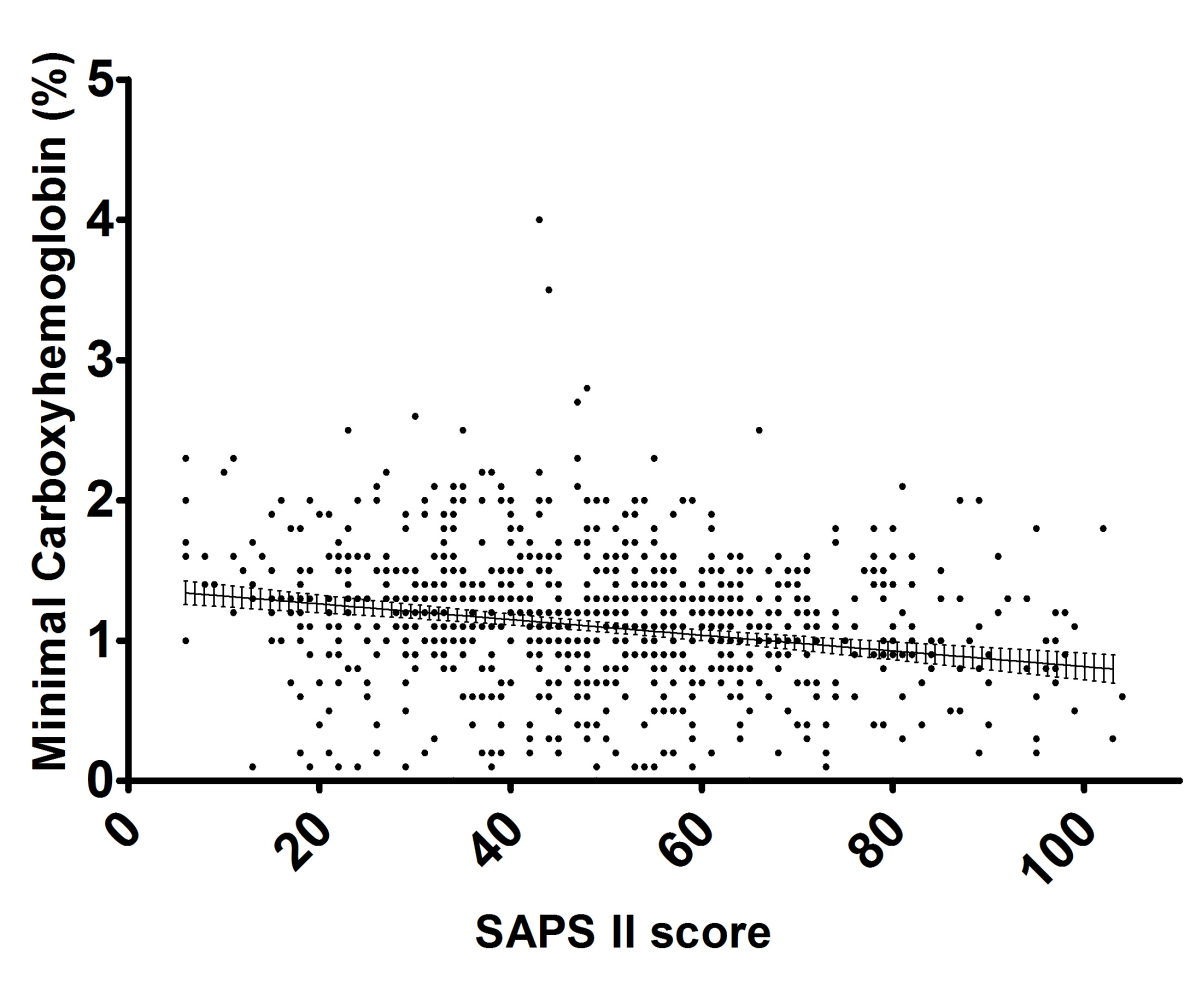
**Correlation between minimal carboxyhemoglobin during the stay on the ICU with SAPS II score**. Pearson's correlation coefficient with 95% confidence intervals: 0.21 (0.14 to 0.27), *P *< 0.0001. The line and error bars (95% confidence intervals) are derived from linear regression.

Minimal COHb was inversely correlated with SAPS II score (Pearson's correlation coefficient with 95% confidence intervals: 0.21 (0.14 to 0.27), *P *< 0.0001) (Figure 3). Therefore, patients who were severely ill on admission had lower minimal levels of COHb during the course of their treatment in the ICU.

### COHb levels andmortality

**Figure 4:**
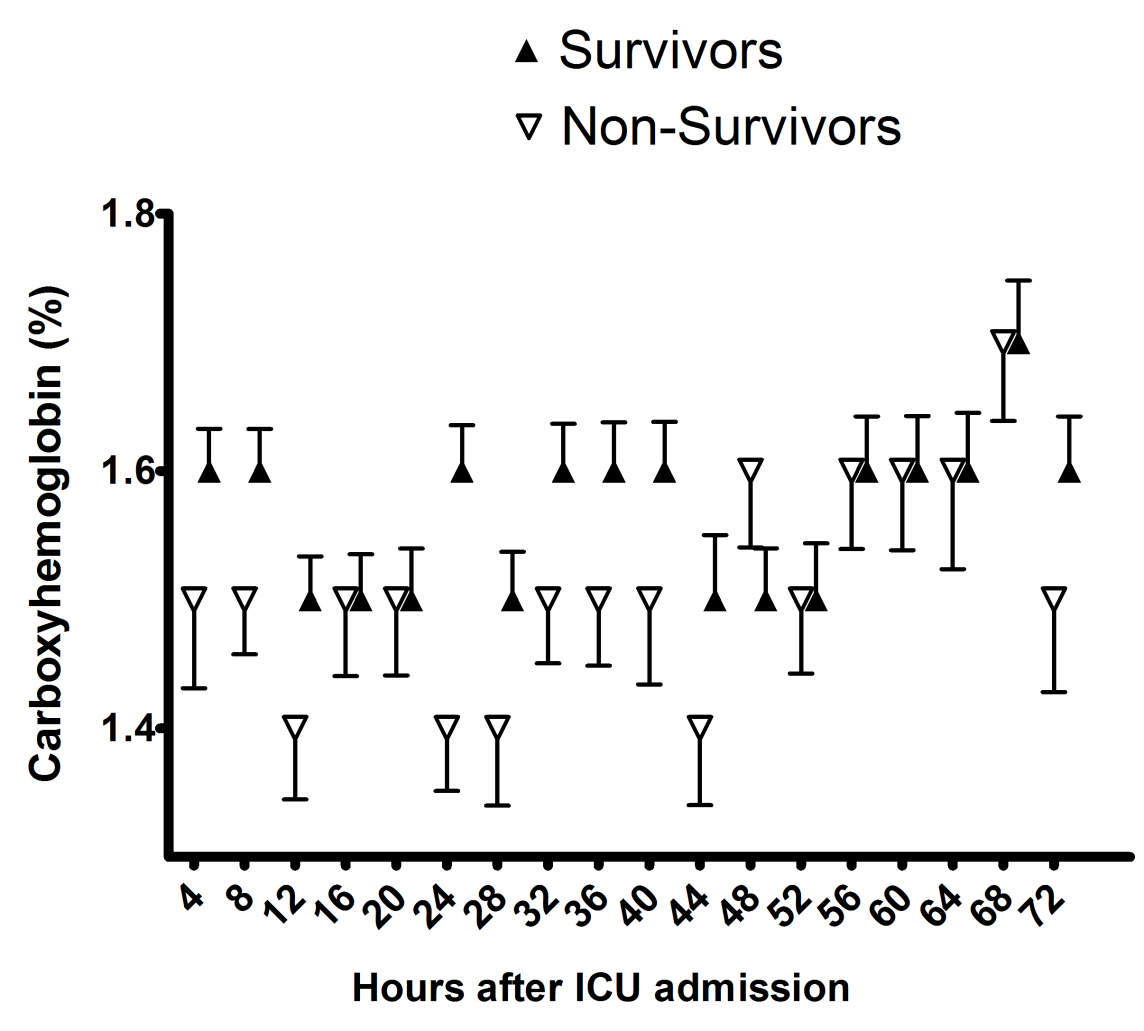
**Mean levels of COHb during the first 72 hours after admission to the ICU**. Empty and full triangles represent non-survivors and survivors, respectively. In order to prevent overlap black triangles were shifted to the right by 1.2 hours.

The non-survivors had a slightly lower minimum COHb level compared to the survivors (0.9%, 0.7% to 1.2% versus 1.2%, 0.9% to 1.5%; *P *= 0.0001); therefore, the patients who died had the lowest COHb levels. In addition, the mean COHb level was marginally lower in the non-survivors (1.5%, 1.2% to 1.8% versus 1.6%, 1.4% to 1.9%; *P *= 0.003). During the first 72 hours after ICU admission average COHb was 0.1% lower in patients who died compared to patients who survived; *P *= 0.003. Differences were most pronounced during the first 48 hours (Figure 4).

The maximum COHb level did not differ between the two groups (survivors 2.1%, 1.7% to 2.6% versus non-survivors 2.1%, 1.5% to 2.5%; *P *= 0.19). The COHb variance was marginally smaller in the survivors compared to the non-survivors (0.0%, 0.0 to 0.1% versus 0.1%, 0.0 to 0.1%; *P *= 0.0001). The COHb area under the curve results were marginally higher in the survivors compared to the non-survivors (1.6%, 1.4% to 1.9% versus 1.5%, 1.2% to 1.8%; *P *= 0.01).

The association between a low minimum COHb level and increased mortality was independent of the severity of illness and the type of organ failure on ICU admission. The results of the multivariate logistic regression are shown in Table 4.

**Table 4:** Logistic regression analysis of the association between minimal COHb level and ICU mortality

	Odds ratio and 95% confidence intervals	***P-***value
**COHb minimum, %**	0.6 (0.4 to 0.8)	0.001
**SAPSII score**	1.0 (1.0 to 1.0)	< 0.001
**Cardiovascular failure on ICU admission, yes/no**	2.2 (1.6 to 3.3)	< 0.001
**Renal failure on ICU admission, yes/no**	1.8 (1.2 to 2.6)	0.003
**Hepatic failure on ICU admission, yes/no**	1.8 (1.2 to 2.7)	0.003

SAPS II score and the type of organ failure on admission were predefined confounders.

## Discussion

Our study shows that the minimum and average COHb levels are marginally higher in medical patients who survive an episode of critical illness compared to non-survivors. The study by Melley *et al. *showed that the minimum COHb level was significantly higher in patients who survived a short ICU stay (median 1.0 (0.9 to 2.8) days) following cardiothoracic surgery compared to patients who died in the ICU [18]. This association was reproduced in our patient cohort of general medical patients who required longer ICU stays (median 6 (3 to 11) days). Furthermore, the maximum COHb level was associated with an increased mortality in the Melley study (*P *< 0.001 in univariate analysis and *P *= 0.08 in multivariate analysis); however, this association was not reproduced in our study.

Our study also shows differences in COHb levels according to admission category with patients admitted with renal and gastrointestinal disease having the highest levels. However, further data would be needed to draw pathophysiological conclusions from this observation. The expected correlation between SAPS II score and COHb level was seen in our study with sicker patients having lower minimal COHb levels.

The strengths of our study include the enrollment of a large patient cohort and being the first study to analyze COHb levels in non-surgical patients who were admitted to a general medical ICU. The limitations of our study include the paucity of data on the smoking status and history of respiratory disease prior to admission, the bilirubin and Hb levels, the inhaled oxygen concentration during the course of the admission and the hospital outcome following ICU discharge. We acknowledge that these limitations need to be taken into account when interpreting our results. Arguably, a strong correlation between arterial COHb, exhaled carbon monoxide (eCO) and total serum bilirubin as indices of heme metabolism exists in critically ill patients [25]; therefore, we would like to suggest that the absence of bilirubin levels does not hamper the quality of our data. On the other hand, we take into account that the lack of data on smoking status might represent a bias in our study. Current smokers have approximately five-fold higher COHb levels than the observed average COHb levels in our study [6]. Of interest, very few of our patients had COHb levels above 2.5%. We speculate that a considerable amount of the smokers' CO has been washed out prior to admission to the ICU as the state of their illness did not allow them to smoke in the hours prior to the admission.

Overall, it remains unclear whether there is an actual causal effect of COHb levels on survival. The anti-inflammatory products of HO-1 induction might be beneficial [26]. Failure to activate protective systems including HO-1 in the face of inflammatory conditions may be deleterious. Indeed, variants of the promoter region of the HO-1 gene with an increased activity were recently found to be associated with a reduced risk for the development of adult respiratory distress syndrome [27].

The observed differences in COHb levels between survivors and non-survivors in our study were statistically significant and indicate that this may be an important pathway. On the other hand, given the relative minuteness of the differences in COHb between the two groups, we have to conclude that COHb does not qualify as a predictive marker for ICU mortality. Nevertheless, we hope that the presented data add to a more complete understanding of the characteristics of COHb in medical intensive care patients.

## Conclusion

Critically ill patients surviving the admission to a medical ICU had slightly higher minimum and marginally higher average COHb levels when compared to non-survivors. Observed margins were minute and do not qualify COHb as a marker for ICU mortality.

## Key messages

• Non-surgical patients surviving critical illness had slightly higher minimum and marginally higher average COHb levels compared to patients who died.

• Observed margins were minute and would not qualify COHb as a marker for ICU mortality.

## Abbreviations

CO, carbon monoxide; COHb, carboxyhemoglobin; COPD, chronic obstructive pulmonary disease; eCO, exhaled carbon monoxide; HO, heme oxygenase (including isoforms HO-1, HO-2); SAPS II, Simplified Acute Physiology Score version II; SOFA, Sepsis-related Organ Failure Assessment.

## Authors' contributions

ASF drafted the manuscript; MW and CZ critically revised the manuscript; GCF was responsible for the conception and design of the study and carried out the statistical analysis. All authors read and approved the final version of the manuscript.

## Competing interests

The authors declare that they have no competing interests.
